# Identification of Resistance to Wet Bubble Disease and Genetic Diversity in Wild and Cultivated Strains of *Agaricus bisporus*

**DOI:** 10.3390/ijms17101568

**Published:** 2016-09-22

**Authors:** Yongping Fu, Xinxin Wang, Dan Li, Yuan Liu, Bing Song, Chunlan Zhang, Qi Wang, Meiyuan Chen, Zhiwu Zhang, Yu Li

**Affiliations:** 1 Engineering Research Center of Chinese Ministry of Education for Edible and Medicinal Fungi, College of Agriculture, Jilin Agricultural University, Changchun 130118, China; yongping.fu@jlau.edu.cn (Y.F.); wxx7418520@163.com (X.W.); junwuzhongxin@126.com (D.L.); liuyuandbl@jlau.edu.cn (Y.L.); song19800123@jlau.edu.cn (B.S.); zhangchun_lan@yeah.net (C.Z.); qwang2003@hotmail.com (Q.W.); 2 The Institute of Edible Fungi, Fujian Academy of Agricultural Sciences, Fuzhou 350014, China; cmy1972@gmail.com; 3 Department of Crop and Soil Sciences, Washington State University, Pullman, Washington, DC 99163, USA

**Keywords:** *Agaricus bisporus*, *Mycogone perniciosa*, highly resistant, SSR, population structure

## Abstract

Outbreaks of wet bubble disease (WBD) caused by *Mycogone* *perniciosa* are increasing across the world and seriously affecting the yield of *Agaricus bisporus*. However, highly WBD-resistant strains are rare. Here, we tested 28 *A. bisporus* strains for WBD resistance by inoculating *M. perniciosa* spore suspension on casing soil, and assessed genetic diversity of these strains using 17 new simple sequence repeat (SSR) markers developed in this study. We found that 10 wild strains originating from the Tibetan Plateau in China were highly WBD-resistant strains, and 13 cultivated strains from six countries were highly susceptible strains. A total of 88 alleles were detected in these 28 strains, and the observed number of alleles per locus ranged from 2 to 8. Cluster and genetic structure analysis results revealed the wild resources from China have a relatively high level of genetic diversity and occur at low level of gene flow and introgression with cultivated strains. Moreover, the wild strains from China potentially have the consensus ancestral genotypes different from the cultivated strains and evolved independently. Therefore, the highly WBD-resistant wild strains from China and newly developed SSR markers could be used as novel sources for WBD-resistant breeding and quantitative trait locus (QTL) mapping of WBD-resistant gene of *A. bisporus*.

## 1. Introduction

*Agaricus bisporus* is widely cultivated throughout the world. Because of its high nutritional value, this mushroom has been an important component of the human diet for more than 200 years. Currently, large-scale commercial production of *A. bisporus* occurs mainly in North America (USA, Canada), Europe (The Netherlands, France) and Asia (China, South Korea, and India) [1,2,3,4]. However, as regions under commercial cultivation continuously expand, the occurrence of diseases caused by fungal, bacterial, and viral pathogens are also increasing. These diseases severely affect the yield and quality of *A. bisporus* [4,5,6].

Wet bubble disease (WBD), one of the most devastating fungal diseases affecting commercial cultivation of *A. bisporus* worldwide, is caused by *Mycogone perniciosa* [7,8,9,10]. Smith et al. (1924) [11] demonstrated that the earliest record of WBD on *A. bisporus* dates back to 1888. The mycopathogen adheres to and penetrates *A. bisporus* during any stage of fruiting body development causing either the characteristic undifferentiated lumps of primordia or the fruiting body’s color changes to brown [7,8,9]. These tumorous bodies are covered with wet bubbles, white and fluffy mycelium, and amber droplets [7,8,9].

China is one of the largest producers of *A. bisporus* (2.0–2.5 million ton per year). In recent years, however, frequent and expanding outbreaks of WBD have occurred across a number of provinces (e.g., Fujian, Sichuan, Hubei, Shanghai, Jiangsu, and Gansu) [12,13]. Moreover, the degree of WBD has become increasingly serious so that WBD is one of the most important factors affecting yield and quality of *A. bisporus.* In commercial cultivation, *M. perniciosa* can commonly cause yield losses of about 15%–30% once WBD develops. However, in severe WBD infection, losses can be as high as 50%–60% or even result in complete crop failures of entire mushroom farms [13,14]. This situation has also been found in India (Kashmir, Maharashtra) [15,16], and Europe (Poland, Serbia) [1,17,18].

Chakravarty (2011) [19] demonstrated the difficulty in controlling many fungal diseases in mushrooms since mushrooms are themselves fungi. Therefore, breeding new advanced cultivars with high WBD resistance is likely the most useful strategy for sustainable control of WBD in *A. bisporus*. However, few studies have evaluated the degree of WBD resistance across multiple *A. bisporus* strains. In 1989, Fletcher reported that all commercial cultivars were susceptible to *M. perniciosa* [4]. Wang et al. (2008) [20] found that three newer Chinese cultivated strains had high resistance to WBD, and six commercial cultivars were highly susceptible.

Therefore, to accelerate the potential for breeding *M. perniciosa* resistance in *A. bisporus*, we designed our study with the following objectives: (1) screen and classify wild and cultivated strains of *A. bisporus* for resistance to *M. perniciosa*; (2) develop simple sequence repeat (SSR) markers for genetic diversity analysis in wild and cultivated strains of *A. bisporus*, favoring their use in breeding programs.

## 2. Results

### 2.1. Identification of WBD Resistance in A. bisporus Strains

**Figure 1:**
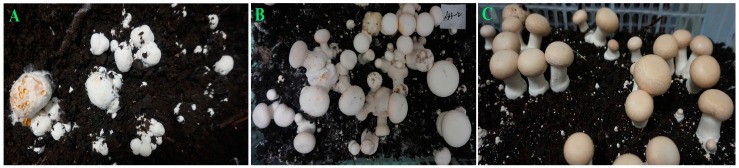
The resistant level of *Agaricus bisporus* to wet bubble disease (WBD). (**A**) Highly susceptible (HS) strain, the mycelia were unable to aggregate into primordia, but instead differentiated into irregular puffballs that exuded amber droplets and released a foul stench; (**B**) moderately resistant (MR) strain, most of the mycelia formed normal primordial and the fruiting bodies, while a part of the fruiting bodies were infected the pathogen with brown spots or amber droplets, and little of the mycelia formed malformed primordial; and (**C**) highly resistant (HR) strain, the mycelia formed normal primordia and fruiting bodies that either without, or with only small, brown spots.

The mycelia of *A. bisporus* aggregated after undergoing an 8–10 days inoculation of WH001 spore suspension. Based on 3 independent tests of WBD assessment, 15 strains of *A. bisporus* (Table 1), including CCMJ1009, CCMJ1013, CCMJ1020, CCMJ1021, CCMJ1022, CCMJ1028, CCMJ1035, CCMJ1037, CCMJ1038, CCMJ1109, CCMJ1110, CCMJ1343, CCMJ1350, CCMJ1352, and CCMJ1384, were found to be highly susceptible to WBD. Among them, 12 strains were cultivated strains, and only 3 strains (CCMJ1110, CCMJ1350, and CCMJ1384) were wild strains. In these strains, the mycelia were unable to aggregate into primordia, but instead differentiated into irregular “puffballs” that exuded amber droplets and released a foul stench (Figure 1A). In contrast, in the control experiments—without inoculation of WH001 spore suspension—the mycelia of these 15 highly susceptible strains were able to normally aggregate into primordia and form fruiting bodies.

We found two strains, including CCMJ1039 and CCMJ1053, were moderately resistant (MR) to WBD. As shown in Figure 1B, most of the mycelia formed normal primordial and the fruiting bodies, while a part of the fruiting bodies were infected the pathogen with brown spots or amber droplets, and little of the mycelia formed malformed primordial.

The 11 highly resistant strains (Table 1), CCMJ1033, CCMJ1106, CCMJ1347, CCMJ1351, CCMJ1360, CCMJ1361, CCMJ1363, CCMJ1369, CCMJ1372, CCMJ1377, and CCMJ1381, formed fruiting bodies either without, or with only small, brown spots (Figure 1C). Of these strains, 10 were wild strains originally collected from China, including 8 wild strains distributed in northwest of Sichuan collected by Qi Wang and provided by Bo Wang, and one distributed in southeast of Tibet and northwest of Yunnan collected by Qi Wang, respectively. Only one strain (C13) was a cultivated strain collected from America. These observations demonstrate the importance of China wild germplasm as sources of resistance to WBD.

According to Koch’s postulates, the fruiting bodies developed visible WBD symptoms 3–5 days after inoculation of the mycopathogen (Figure S1). These symptoms were consistent with the symptoms observed at the Edible Mushroom Base of our center. In contrast, the fruiting bodies in the control treatments (without inoculation) grew normally. The internal transcribed spacer (ITS) sequencing results of the isolated pathogen were 100% homologous to relevant nucleic acid sequences of *M. perniciosa* strains in the NCBI dataset. Thus, Koch’s postulates confirmed that the pathogen reisolated from the diseased primordia and fruiting bodies of *A. bisporus* was the *M. perniciosa* strain used in the assessment.

### 2.2. Assessment of Genetic Diversity in A. bisporus Using New SSR Markers

Based on the sequenced genome of *A. bisporus*, we developed 1838 new SSR primers for the SSR locus (Table S1). Of these primers, 1188 (64.6%) were for dinucleotide repeats (DNR), 606 (33.0%) were for trinucleotide repeats (TNR), 20 (1.1%) were for tetranucleotide repeats (TTNR), 10 (0.5%) were for pentanucleotide repeats (PNR), and 14 (0.8%) were for hexanucleotide repeats (HNR). Two hundred of the newly developed primers were screened for polymorphism in *A. bisporus* (Table S2). Among them, 17 SSR primers (8.5%) did not amplify a band, 39 SSR primers (19.5%) amplified one band, and 144 SSR primers (72%) exhibited polymorphism (Table S2). These 144 polymorphic primers can be used for subsequent genetic diversity studies in *A. bisporus*.

We selected 17 polymorphic, clear and stable SSR markers to assess the genetic diversity of these 28 *A. bisporus* strains. Genetic parameters of the 17 polymorphic SSR markers are shown in Table 2. In total, 88 alleles were detected across all 28 strains of *A. bisporus*. The observed number of alleles per locus (Na) ranged from 2 (AbSSR17) to 8 (AbSSR17), with an average value of 5.176 per locus. The observed heterozygosity (Ho), expected heterozygosity (He) and I value were 0.000–0.786 (mean = 0.319), 0.394–0.819 (mean = 0.6807), and 0.598–1.905 (mean = 1.306), respectively. The polymorphism information content (PIC) ranged from 0.325 to 0.816 with an average value of 0.618. These results indicated a high level of genetic diversity in the 28 *A. bisporus* strains.

**Figure 2:**
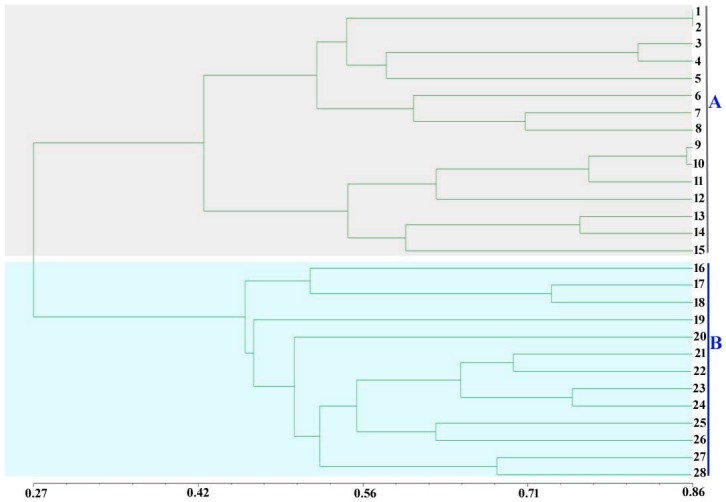
Unweighted pair-group method with arithmetic (UPGMA) dendrogram of 28 *Agaricus bisporus* strains constructed by using genetic similarity analysis based on 17 new SSR markers. These strains are clustered into two groups: A (15 strains) and B (13 strains) at genetic similarity (GS) coefficient value 0.27. Group A are cultivated strains, including 1-CCMJ1009, 2-CCMJ1021, 3-CCMJ1020, 4-CCMJ1038, 5-CCMJ1033, 6-CCMJ1037, 7-CCMJ1039, 8-CCMJ1053, 9-CCMJ1013, 10-CCMJ1022, 11-CCMJ1028, 12-CCMJ1035, 13-CCMJ1343, 14-CCMJ1109, and 15-CCMJ1352. Group B are wild strains, including 16-CCMJ1106, 17-CCMJ1350, 18-CCMJ1361, 19-CCMJ1351, 20-CCMJ1377, 21-CCMJ1347, 22-CCMJ1360, 23-CCMJ1363, 24-CCMJ1369, 25-CCMJ1381, 26-CCMJ1384, 27-CCMJ1110, and 28-CCMJ1372.

A dendrogram (Figure 2) was constructed using unweighted pair-group method with arithmetic (UPGMA) based on genetic similarity (GS) coefficient value of DICE for the 28 *A. bisporus* strains. The collections were clustered into two groups at GS coefficient value 0.27. The result was relative to the strain types of *A. bisporus*. For example, Group A contained 15 strains that were cultivated strains from six countries; Group B consisted of 13 strains that were wild strains originating from China. Group A was further subdivided in Subgroup A1 and A2 at GS coefficient value 0.42. The highly WBD-resistant cultivated strains (C13) and moderately resistant cultivated strains (126 and M-1) were in Subgroup A1. These results indicated the high genetic variation between Group A (cultivated strains) and Group B (wild strains originated from China). Therefore, we suggest that the newly developed SSR markers in our work could be used as robust molecular markers for population genetic studies in *A. bisporus*.

The 28 *A. bisporus* strains were assigned into two separate subpopulations (wild and cultivated populations) based on the dataset of 17 SSR markers using GenAIEx. The analysis results of the two subpopulations are shown in Table S3. The Nei’s genetic distance and genetic identity of the two subpopulations were 0.717 and 0.488, respectively. Based on codominant allelic distance matrix for calculation of *Fst* (within individual analysis suppressed), the result of analysis of molecular variance (AMOVA) showed that the genetic differentiation among the two subpopulations was 25%, while within subpopulations was 75%. The value of gene flow (Nm) based on *Fst* value among the two subpopulations was 0.738. In addition, the genetic differentiation coefficient (Gst) among the two subpopulations was 0.147, which also indicated most of variance occurred within each subpopulation.

**Figure 3:**
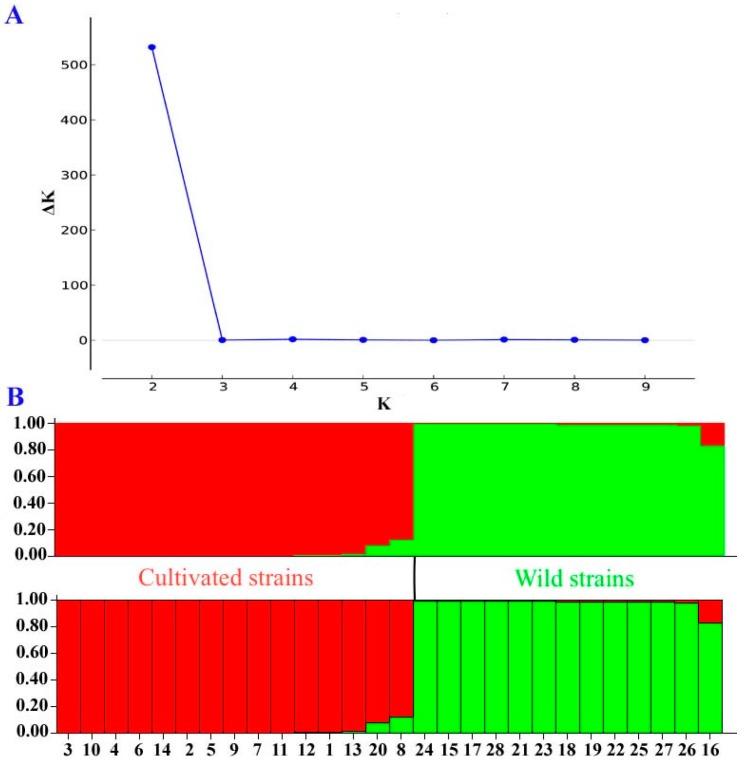
Population structure of 28 *Agaricus bisporus* strains according to STRUCTURE analysis at *K* = 2, based on the 17 SSR markers. (**A**) Measure of ΔK for each K value; (**B**) A bar represents a sample. Red color represents cultivated strain population. Green color represents wild strain population. From left to right on the bottom the samples are, in order, CCMJ1020, CCMJ1038, CCMJ1021, CCMJ1028, CCMJ1109, CCMJ1013, CCMJ1022, CCMJ1037, CCMJ1033, CCMJ1039, CCMJ1053, CCMJ1009, CCMJ1343, CCMJ1352, CCMJ1035, CCMJ1369, CCMJ1106, CCMJ1347, CCMJ1384, CCMJ1360, CCMJ1363, CCMJ1350, CCMJ1351, CCMJ1361, CCMJ1372, CCMJ1381, CCMJ1377, and CCMJ1110.

The population structure of *A. bisporus* was also inferred with the dataset of 17 SSR markers using STRUCTURE software. When K = 2, ΔK = 86 is the maximum peak, suggested K = 2 as the optimal number of populations (Figure 3). As can be clearly seen in Figure 3, the 28 strains of *A. bisporus* were mainly divided into two subpopulations that separated wild strains of China from cultivated strains. The result was identical with the obtained UPGMA dendrogram and GenAIEx, and supported the accuracy of the clustering.

## 3. Discussion

Breeding for tolerance to WBD has historically been problematic due to scarce genotypes of *A. bisporus* resistant to WBD. Until now, less than five strains with WBD resistance have been identified [20], and the genetic basis underlying the resistance has not been described. Currently, most cultivated strains of *A. bisporus* have been bred by North America, Europe, and China [21,22]. Moreover, wild resources of *A. bisporus* encompass more genetic diversity than commercial cultivars [23,24,25,26]. Therefore, to ensure a comprehensive analysis of *A. bisporus* resistance to WBD, we identified cultivated strains from the abovementioned countries and new resources of wild strains. Furthermore, we also adopted sampling considering a high degree of variation in morphological features (Figure S2), such as the cap colors ranging from white to brown, caps being smooth to scaly, and stems being short to long. In addition, we collected all cultivated strains of *A. bisporus* from Fujian in China due to 80% cultivated strains having been bred by Edible Fungi Institute of Fujian Academy of Agricultural Sciences in the past 20 years; for example, As2796 (CCMJ1013) is the major cultivar used in different provinces of China.

Previous studies showed that the wild resources of *A. bisporus* in China are mainly found in the Tibetan Plateau, including most of the Tibet Autonomous Region and Qinghai province, and part of Sichuan, Gansu, Xinjiang, and Yunnan provinces [22,27,28]. In our study, we collected more wild strains in northwest of Sichuan, and a wild strain from Tibet, Yunnan, and Wutai Mountain in Shanxi, respectively. Therefore, we suggest the Tibetan Plateau in China could cover more wild resources of *A. bisporus* and is also the world center of *A. bisporus* origin and genetic diversity. We speculated that the main reason may be that these contiguous regions, such as Sichuan and Tibet, have less human interference, similar natural conditions, airborne spores, and gene flow.

In our study, we obtained similar results compared with previous studies, in which most of the commercially cultivated strains, including Horst U1 and their derivative (such as A15), were susceptible to *M. perniciosa* [4,20]. Fortunately, we also obtained 10 wild strains originating from the Tibetan Plateau in China that were highly resistant to WBD. The main reason may be that these wild resources have adapted to ecological habitats of the Tibetan Plateau, and have accumulated considerable genetic variation to increase stress resistance. However, except for one wild strain distributed in Wutai Mountain in Shanxi, two wild strains from Sichuan were highly sensitive to WBD. These variations in phenotype also illustrated that there was rich genetic diversity among the tested wild resources in the Tibetan Plateau. We need to further explore the correlation between the phenotypes and genotypes. Therefore, these new wild germplasm of highly WBD-resistant strains for *A. bisporus* could be valuable resources of stable resistance for breeding and genetic studies. Breeders could select suitable ecotype strains to cross according to the WBD-identified result.

Some research indicated that the brown cap color strain of *A. bisporus* was less susceptible than white strains to bacterial and green mold [5,29,30]. In our study, the results also showed that 11 out of 13 brown strains of *A. bisporus* were highly resistant to WBD, while only 1 out of 15 white strains was resistant to WBD. We speculated that the WBD-resistant associated gene of *A. bisporus* might be closely linked to natural cap color genes. We suggested that the brown wild brown highly WBD-resistant strains, as new genetic resources, represent a great potential for breeders to improve WBD-resistance and quantitative trait locus (QTL) mapping the resistant genes in *A. bisporus*.

SSR markers have proven to be simple, useful, and reliable for germplasm identification, genetic diversity studies, linkage map construction, and molecular marker-assisted selection in breeding [31,32,33,34,35]. Until now, only 33 useful SSR markers have been developed for *A. bisporus* [36]. Therefore, there is an urgent need to develop more highly polymorphic and user-friendly SSR markers for genetic studies of *A. bisporus.* In this study, we provide 144 new informative SSR markers as a valuable resource that can be used for further unambiguous identification of genotypes, and the molecular phylogeny and evolution in *A. bisporus*. We further indicated that 17 newly developed SSR markers are suitable markers that can differentiate genetic variability between wild strains originating from China and cultivated strains, and identify genetic relatedness within wild and closely related cultivated strains (Horst U1 and their derivatives) of *A. bisporus*. In addition, previous studies verified that wide genetic variability among the wild strains originating from Europe, America, China, and Iran [22,23,25,26,27,31]. According to the analysis results using the 17 newly developed SSR markers, we confirmed the inherent genetic variability of wild strains originating from China, and found 25% of SSR alleles that were lacking in cultivated strains. Therefore, we speculated the wild resources of *A. bisporus* from the Tibetan Plateau in China have specific genetic groups and maintain a relatively high level of genetic diversity. We also provide the new or rare alleles to breeding gene pools of *A. bisporus*. The use of wild strains from China and new SSR markers should facilitate broadening the genetic base and WBD tolerance improvement, and the QTL mapping of highly WBD-resistant genes.

The population structure results of the 28 *A. bisporus* strains indicated that wild subpopulation originating from China and cultivated subpopulation come from different ancestries. The results also implied a low level of introgression occurred between two subpopulations due to the geographic distance. Among the two subpopulations, cultivated subpopulation was suspected to have the consensus ancestral genotypes with the European population(s), while wild subpopulation was suspected to have the consensus ancestral genotypes originating from China and potentially evolved independently. A previous report [37] indicated that the European population(s) was principally known crossing the cultivated strains from Europe and one American strain. In our studies, within cultivated *A. bisporus* strains, we suggested that European and American strains have experienced high-level introgression due to hybridization and gene flow. Based on the values Fst (0.253) and Gst (0.147), we also found a low level of gene flow between two subpopulations, which may be led to genetic differentiation among the two subpopulations. We think that these genetic differentiations increase the genetic heterogeneity in wild *A. bisporus* resources and improve the ability to adapt to the Tibetan Plateau environment. Thus, these genetic diversity and genetic structure analyses provide the insights into the origin and evolution of wild *A. bisporus* resources mainly distributed in the Tibetan Plateau of China, and are valuable for defining new gene pools and developing breeding programs for *A. bisporus*.

In summary, the wild highly WBD-resistant strains of *A. bisporus* originating from China have rich genetic variance, which can be used directly as the parents for development of new highly WBD-resistant hybrid cultivars. The newly developed SSR markers in this work can be useful tool to assess population structure and genetic diversity in *A. bisporus*, and identify molecular markers for marker-assisted selection.

## 4. Materials and Methods

### 4.1. M. perniciosa and A. bisporus Strains

Strain WH001 of *M. perniciosa* was isolated from diseased fruiting bodies of *A. bisporus* from a mushroom farm in Wuhan province (China) in a previous study [13]. Twenty-eight *A. bisporus* strains were used to evaluate WBD resistance (Table 1). Thirteen wild strains originated from Sichuan, Yunnan, and Shanxi provinces, and from Xizang Autonomous Region in China. Fifteen cultivated strains were collected from Germany, England, The Netherlands, Spain, USA, and China (Fujian province). All strains of *M. perniciosa* and *A. bisporus* used in this study are maintained in Engineering Research Center of Chinese Ministry of Education for Edible and Medicinal Fungi of Jilin Agricultural University, China.

### 4.2. Evaluation of A. bisporus Strains for Resistance to WBD under Controlled Environmental Conditions

The screening of *A. bisporus* for resistance against WBD experiments were conducted on September 2014, March 2015, and September 2015, respectively. Each time, all 28 *A. bisporus* strains were cultivated with the same batch of compost and casing soil (4 cm applied to cover the compost) in a controlled incubation and fruiting room at the Edible Mushroom Base of Jilin Agricultural University, China. Cultivation of *A. bisporus* was performed in a plastic tray (45 × 55 × 30 cm) containing 7.5 kg of compost (wheat straw, cattle manure, gypsum, and soy meal). Each plastic tray was inoculated with 80 g of spawns and incubated at 25 °C (RH 95%; 3500 ppm CO_2_) for a period of 15–20 days. Eighteen trays were inoculated for each strain; nine for disease assessment, the other nine as control. The casing soil (peat moss) was sterilized by formaldehyde fumigation for 24 h and left for another 24 h to eliminate any residual formaldehyde prior to the artificial inoculation of WH001 strain of *M. perniciosa* on trays after spawn run.

Spore suspensions were obtained from the WH001 strain of *M. perniciosa* grown on Potato Dextrose Agar (PDA) for 6 days at 25 °C. Spores were collected and diluted with sterile distilled water (SDW) at a concentration of 1 × 10^5^ cfu/mL. Spore numbers were determined by plate count for colonies using the mean of three counts. Fifty milliliters of the spore suspension (1 × 10^5^ cfu) was dripped onto the casing layer. After *A. bisporus* mycelia permeated the casing layer (after 16–17 days casing), the room temperature was cooled to 16~18 °C. For each *A. bisporus* strain, nine incubated trays were dripped with the spore suspension, and another nine trays were dripped with 50 mL of SDW as control.

The resistance level of WBD of each *A. bisporus* strain was classified according to the visual assessment of WBD severity within the primordia and fruiting bodies, as follows: (1) highly susceptible (HS) = the mycelia form malformed primordia or fail to form normal primordial in the incubated trays; (2) moderately resistant (MR) = few of the mycelia form malformed primordia, and most of the mycelia form normal primordial and the fruiting bodies, while a part of the fruiting bodies are infected with the pathogen; and (3) highly resistant (HR) = formed normal primordia and fruiting bodies, and nearly all fruiting bodies show no WBD infection.

### 4.3. Identification of the Pathogen Using Koch’s Postulates

Koch’s postulates were performed to verify whether the WBD symptoms observed were indeed caused by the *M. perniciosa* WH001 strain used during the evaluation. The pathogen strains from the diseased primordia and fruiting bodies were reisolated and cultured on PDA. Genomic DNA of the pathogen was isolated using the DNA Extraction Kit (KANGWEI, Beijing, China) following manufacturer instructions. Internal transcribed spacer (ITS) gene was also used to identify the pathogen strains by PCR amplification and sequencing. ITS primers and PCR amplification conditions were the same as White et al. (1990) [38]. PCR reaction volume was 25 μL, containing 2.5 μL 10× buffer, 2.5 μL MgCl_2_ (25 μM), 0.6 μL of dNTPs (10 mM) (Thermo Scientific, Waltham, MA, USA), 0.2 μL Taq DNA polymerase (Thermo Scientific, Waltham, MA, USA), 1 μL each primer (2.0 μM), 1 μL DNA (20 ng/μL), and 16.2 μL ddH_2_O. PCR products were electrophoresed in 1% agarose stained with SYBR^®^ safe DNA gel stain (Thermo Scientific, Waltham, MA, USA), and then sequenced in Sangon Biotech Co., Ltd. (Shanghai, China). Using BLAST, these sequencing results were compared to the sequence of *M. perniciosa* in the NCBI dataset.

### 4.4. Development of SSR Markers for A. bisporus

The H97 genome sequences (version 2.0) of *A. bisporus* (http://genome.jgi-psf.org/.) were used for scanning microsatellite loci using MISA (http://pgrc.ipk-gatersleben.de/tools.php). Primer design of microsatellite motifs was performed using Primer 3 software (Whitehead Institute, Cambridge, MA, USA).

A total of 200 SSR primers (Table S2) were selected and synthesized by Sangon Biotech Co., Ltd. (Shanghai, China). Five wild and five cultivated strains were used to evaluate polymorphism (Table S4). The methods of Genomic DNA isolation, PCR amplification reaction and condition, and polyacrylamide gel electrophoresis were same as Fu et al. (2016) [39], except for the annealing temperature using 56 °C in PCR amplification condition.

### 4.5. Identification of Genetic Diversity for the 28 A. bisporus Strains

Seventeen SSR markers (Table 2) were used for the identification of genetic diversity in the 28 *A. bisporus* strains. Amplified DNA fragments of SSR markers were scored as present (1) or absent (0). CURVE and GenAIEx6.502 software were used to calculate genetic diversity parameters for each SSR locus. NTSYS-pc software (Version 2.1, Applied Biostatistics, Setauket, NY, USA) was used to construct a dendrogram using unweighted pair-group mean algorithm (UPGMA) cluster. STRUCTURE software (Version 2.3.4, Stanford University, Stanford, CA, USA) and Structure Harvester program [40,41] were used to examine population genetic structure.

## 5. Conclusions

In this study, a comprehensive analysis of *A. bisporus* strains resistance to WBD is presented, with the following results: (1) Ten wild strains originating from China were highly resistant to WBD, and most of the cultivated strains were highly susceptible; (2) there is a high level of genetic diversity in these 28 *A. bisporus* strains based on 17 new SSR markers developed from the sequenced genome of *A. bisporus*. These results provide a good foundation for further mushroom WBD-resistant breeding and, potentially, a highly effective method to help control the rapid spread of WBD.

## Figures and Tables

**Table 1:** Strains of *A. bisporus* used for WBD assessment and genetic diversity.

Strain Name	Original Reference	Origin	Pileus Color	Strain Types	Resistance Level ^a^
CCMJ1009	A15	America	White	Cultivated strain	HS
CCMJ1013	As2796	Fujian, China	White	Cultivated strain	HS
CCMJ1020	ZA	Germany	White	Cultivated strain	HS
CCMJ1021	S130A	America	White	Cultivated strain	HS
CCMJ1022	As4580	Fujian, China	White	Cultivated strain	HS
CCMJ1028	S46	Fujian, China	White	Cultivated strain	HS
CCMJ1033	C13	America	Brown	Cultivated strain	HR
CCMJ1035	72	America	White	Cultivated strain	HS
CCMJ1037	U1	The Netherlands	White	Cultivated strain	HS
CCMJ1038	PSU310	America	White	Cultivated strain	HS
CCMJ1039	126	The Netherlands	White	Cultivated strain	MR
CCMJ1053	M-1	Spain	White	Cultivated strain	MR
CCMJ1106	2094	Tibet, China	Brown	Wild stain	HR
CCMJ1109	Ag23	England	White	Cultivated strain	HS
CCMJ1110	Dashan I	Shanxi, China	Brown	Wild stain	HS
CCMJ1343	W192	Fujian, China	White	Cultivated strain	HS
CCMJ1347	T12387	Yunnan, China	White	Wild stain	HR
CCMJ1350	W1	Sichuan, China	Brown	Wild stain	HS
CCMJ1351	W2	Sichuan, China	Brown	Wild stain	HR
CCMJ1352	A12	America	White	Cultivated strain	HS
CCMJ1360	W3	Sichuan, China	Brown	Wild stain	HR
CCMJ1361	W4	Sichuan, China	Brown	Wild stain	HR
CCMJ1363	W5	Sichuan, China	Brown	Wild stain	HR
CCMJ1369	W6	Sichuan, China	Brown	Wild stain	HR
CCMJ1372	W7	Sichuan, China	Brown	Wild stain	HR
CCMJ1377	W8	Sichuan, China	Brown	Wild stain	HR
CCMJ1381	W9	Sichuan, China	Brown	Wild stain	HR
CCMJ1384	W10	Sichuan, China	Brown	Wild stain	HS

^a^ Highly susceptible (HS), moderately resistant (MR), highly resistant (HR).

**Table 2:** Characteristics of the 17 polymorphic simple sequence repeats (SSR) markers of *A. bisporus* used in this study.

Locus	SSR motif	Primer Sequence (5′–3′)	Ta (°C)	Na	Ne	Ho	He	PIC	I
AbSSR05	(GATGAG)_6_	F-CTCTGGGATATGGACGAGGA R-CCTCTTCACCTTGACCCTCA	56	5	3.2197	0.5	0.702	0.635	1.3177
AbSSR08	(TGG)_8_	F-GTAATGCTCCCGCTGTTGAT R-TCCGCTGTTCTTCCAACTCT	56	4	2.3939	0.107	0.593	0.531	1.0785
AbSSR10	(CCA)_8_	F-GAAGAATCACGGGTGAAGGA R-GAGGGCGATGTGACAGTTTT	56	8	6.1455	0.423	0.854	0.816	1.905
AbSSR14	(TACC)_6_	F-GGCAATCGGAAAGAACAAAA R-GCAGAGAACCATCCTCAACG	56	7	4.2757	0.333	0.781	0.73	1.6081
AbSSR15	(TA)_6_	F-GACTGCCTGATTGACGGATT R-TCCGACTCCGACATCCTATC	56	6	4.2609	0.393	0.779	0.729	1.5613
AbSSR17	(CA)_6_	F-GGACGAACTTATGCCGTGTT R-GGCACAGCCTGAGAGAGAAG	56	2	1.6897	0	0.416	0.325	0.5983
AbSSR18	(GA)_7_	F-CTCGAGTCGACGAAGGAAAC R-TCCTCGGTTTCGACTGTACC	56	5	2.9418	0.786	0.672	0.611	1.2567
AbSSR28	(TC)_12_	F-TGTCTGGTTTTGCTCACGTC R-TCAGCACACTTAATCGCACA	56	4	2.5331	0.321	0.616	0.539	1.058
AbSSR47	(CA)_8_	F-CATCGGAATCTGAGCTGTCA R-TGTGTCAAAAGTGGGTCCAA	56	4	3.0519	0.308	0.686	0.607	1.1893
AbSSR52	(CAT)_6_	F-TGGCTCTTTACAGCCTTGGT R-TGCAGATGTGGTAGGAGTTTTG	56	6	3.3212	0.556	0.712	0.654	1.4004
AbSSR75	(CAA)_7_	F-CGTCCAACATCAACGTCAAC R-GTGTACATCCCCTCGTCGTC	56	6	5.0827	0.5	0.819	0.775	1.7012
AbSSR85	(CGT)_5_	F-GACTGTTGACGTTTCGGGTT R-CAACGATGACCCGTTTTCTT	56	3	2.1189	0	0.538	0.421	0.8165
AbSSR87	(CCT)_6_	F-CAGTCGCACTCGAAATCGTA R-TTGTTGAGTGAGGCATCGAG	56	5	1.6309	0.308	0.394	0.361	0.7984
AbSSR89	(CAT)_7_	F-GATAGCTCCTGGTCACCGTC R-CTGGCTTCAAGAAGCGTACC	56	6	4.0412	0.214	0.766	0.713	1.5111
AbSSR111	(GAG)_12_	F-TGTCGATTGCGTCTTCTTTG R-CGCCTCGTTTCTCTACTTCG	56	7	4.2151	0.536	0.777	0.728	1.6399
AbSSR112	(CAC)_5_	F-TCACCCTCACTCAAACTCCC R-TCTCATCCGGTTCAACAACA	56	3	2.8403	0	0.661	0.573	1.0695
AbSSR159	(GAA)_6_	F-CGACCCATCATCAACTTCCT R-AACGAGGGAAAGGTCGATTT	56	7	4.8246	0.143	0.807	0.763	1.697
Mean				5.1765	3.4463	0.3193	0.6807	0.6183	1.3063

Ta: annealing temperature; Na: observed number of alleles per locus; Ne: effective number of alleles per locus; Ho: observed heterozygosity; He: expected heterozygosity; PIC: Mean polymorphic information content; I: Shannon’s information index.

## Supplementary Materials

Supplementary materials can be found at www.mdpi.com/1422-0067/17/10/1568/s1.

## Abbreviations

WBD	Wet Bubble Disease
SSR	Simple Sequence Repeat
HS	High Susceptible
MR	Middle Resistant
HR	High Resistant
ITS	Internal Transcribed Spacers
PCR	Polymerase Chain Reaction
QTL	Quantitative Trait Locus
MISA	MIcroSAtellite
