# Supplementary material for: Identification of Resistance to Wet Bubble Disease and Genetic Diversity in Wild and Cultivated Strains of Agaricus bisporus
Source: Int J Mol Sci. 2016 Sep 22;17(10):1568. doi: 10.3390/ijms17101568 (PMC5085624; doi:10.3390/ijms17101568)
Supplement: Supplementary file 1 [file ijms-17-01568-s001.zip › ijms-142570-Supplementary Materials/ijms-142570-supplementary.pdf]

Supplementary Materials: Identification of Resistance to Wet Bubble Disease and Genetic Diversity in Wild and Cultivated Strains of *Agaricus bisporus*

Yongping Fu, Xinxin Wang, Dan Li, Yuan Liu, Bing Song, Chunlan Zhang, Qi Wang, Meiyuan Chen, Zhiwu Zhang and Yu Li

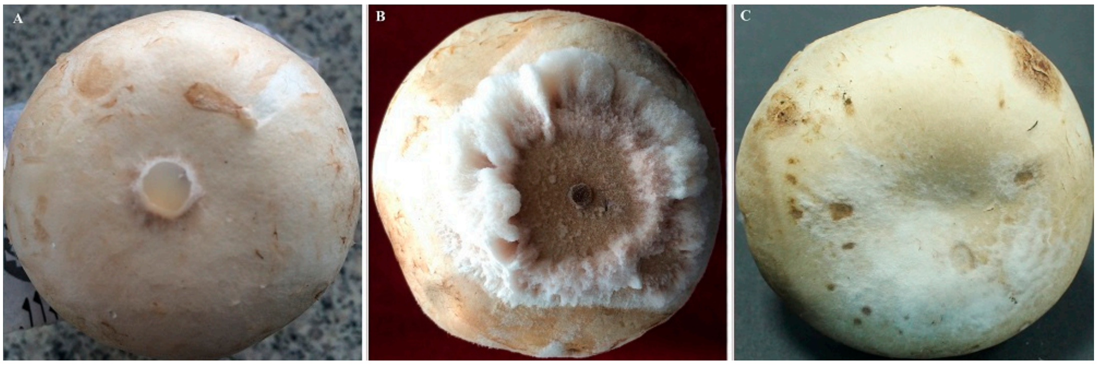

Figure S1. Identification results of Koch's postulates. (**A**) The fruiting bodies were inoculated the mycopathogen on the second day; (**B**) The fruiting bodies developed visible WBD symptoms 7 days after inoculation of the mycopathogen; (**C**) The fruiting bodies did not inoculate the mycopathogen after 7 days.

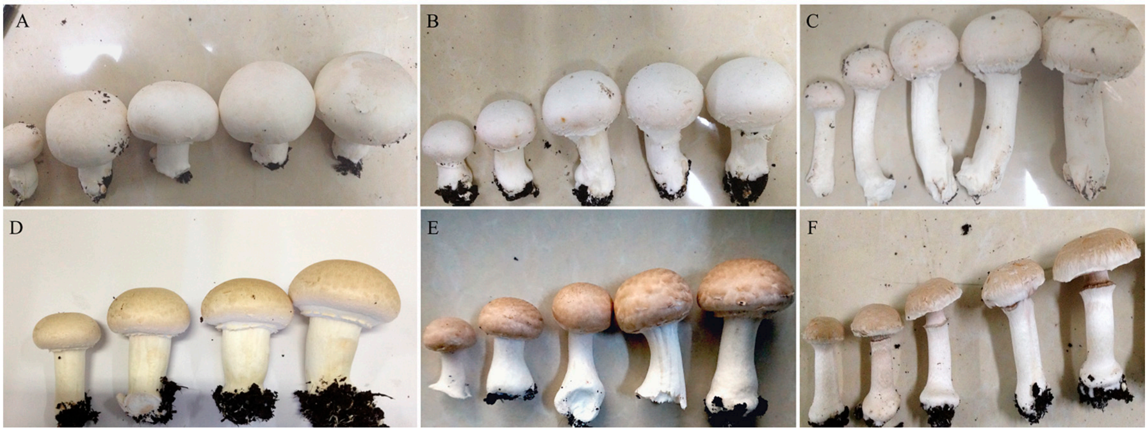

Figure S2. The selected *Agaricus bisporus* strains show a high degree of variation according to the morphology. (**A–F**) The cap colors range from white to brown, caps are smooth to scaly, stems are short to long.
